# Machine learning based radiomics approach for outcome prediction of meningioma – a systematic review

**DOI:** 10.12688/f1000research.162306.1

**Published:** 2025-03-25

**Authors:** Saroh S, Saikiran Pendem, Prakashini K, Shailesh Nayak S, Girish R Menon, Priyanka -, Divya B

**Affiliations:** 1 Department of Medical Imaging Technology, Manipal College of Health Professions, Manipal Academy of Higher Education, Manipal, Karnataka, 576104, India; 2 Department of Radio Diagnosis and Imaging, Kasturba Medical College, Manipal Academy of Higher Education, Manipal, Karnataka, 576104, India; 3 Department of Neurosurgery, Kasturba Medical College, Manipal Academy of Higher Education, Manipal, Karnataka, 576104, India; 4 Department of Electronics and Communication Engineering, Manipal institute of Technology, Manipal Academy of Higher Education, Manipal, Karnataka, 576104, India

**Keywords:** Meningioma, recurrence, outcome prediction, overall survival analysis, machine learning, radiomic features

## Abstract

**Introduction:**

Meningioma is the most common brain tumor in adults. Magnetic resonance imaging (MRI) is the preferred imaging modality for assessing tumor outcomes. Radiomics, an advanced imaging technique, assesses tumor heterogeneity and identifies predictive markers, offering a non-invasive alternative to biopsies. Machine learning (ML) based radiomics models enhances diagnostic and prognostic accuracy of tumors. Comprehensive review on ML-based radiomics models for predicting meningioma recurrence and survival are lacking. Hence, the aim of the study is to summarize the performance measures of ML based radiomics models in the prediction of outcomes such as progression/recurrence (P/R) and overall survival analysis of meningioma.

**Methods:**

Data bases such as Scopus, Web of Science, PubMed, and Embase were used to conduct a literature search in order to find pertinent original articles that concentrated on meningioma outcome prediction. PRISMA (Preferred reporting items for systematic reviews and meta-analysis) recommendations were used to extract data from selected studies.

**Results:**

Eight articles were included in the study. MRI Radiomics-based models combined with clinical and pathological data showed strong predictive performance for meningioma recurrence. A decision tree model achieved 90% accuracy, outperforming an apparent diffusion coefficient (ADC) based model (83%). A support vector machine (SVM) model reached an area under curve (AUC) of 0.80 with radiomic features, improving to 0.88 with ADC integration. A combined clinico-pathological radiomics model (CPRM) achieved an AUC of 0.88 in testing. Key predictors of recurrence include ADC values, radiomic scores, ki-67 index, and Simpson grading. For predicting overall survival analysis of meningioma, the combined clinicopathological and radiomic features achieved an AUC of 0.78.

**Conclusion:**

Integrating radiomics with clinical and pathological data through ML models greatly improved the outcome prediction for meningioma. These ML models surpass conventional MRI in predicting meningioma recurrence and aggressiveness, providing crucial insights for personalized treatment and surgical planning.

Meningioma is the most prevalent type of cancer identified in the brain which is mostly affecting adults. Approximately 7.86 individuals per 100,000 are diagnosed annually with meningioma globally, which is significantly attributed to the credibility of advancements in imaging technology for tumour diagnosis in the last three decades. ^1– 2^ The World Health Organization (WHO) has classified meningiomas into three distinct grades of which the Grade I meningiomas are represented by their slow growth, while Grade II meningiomas are categorised as atypical and Grade III meningiomas represent the most fatal form, being malignant in nature. Thus, both Grade II and Grade III meningiomas display a high rate of recurrence following routine treatment, which may obviously end up with a reduced life expectancy. These facts shed light on the early detection and subsequent treatments which are quite mandatory for the precise diagnosis of meningiomas. ^3– 5^ After meningioma surgery, overall survival at 5 and 10 years respectively were 92.6% and 85%. Survival is prolonged in women, younger adults and those with convexity and benign tumor. ^6^

Magnetic Resonance Imaging (MRI) is an emerging and most effective method for detecting meningiomas and monitoring specific medical conditions. Conventional MR imaging findings such as tumor size, bone invasion, and parasagittal location have all been identified as important imaging parameters related to progression/recurrence (P/R) in meningiomas. ^7– 9^

Radiomics incorporates a range of advanced techniques used to conduct a comprehensive analysis of medical images. Radiomics is primarily intended to analyse the characteristics of tissues and lesions, with respect to their shape and heterogeneity, and monitor changes over a period through serial imaging, during treatment period. ^10– 12^ Examining tissue heterogeneity is crucial, as genomic studies highlight its role as prognostic factor for survival and a challenge in cancer management. Radiomic features strongly correlate with cellular heterogeneity indices. Unlike traditional biopsies, which sample limited tumour areas, radiomics enables heterogeneity assessment across the entire tumour. Leveraging large datasets, radiomics identifies unrecognized markers and patterns related to disease progression and treatment. Additionally, integrating radiomic data with clinical, histological, genomic, and other datasets through machine learning enhances its diagnostic and prognostic utility. ^13– 18^ There are no reviews conducted on evaluating the performance of machine learning (ML) based radiomic models in the prediction of recurrence and overall survival for meningioma. Hence, the aim of the study is to summarize the performance of the ML based radiomics models in the prediction of outcomes such as P/R and overall survival analysis for meningioma.

## Methods

PRISMA ^19^ (Preferred reporting items for systematic reviews and meta-analysis) standards were followed in conducting the review. (PRISMA checklist- Extended data)

### Literature search strategy

Using databases such as Scopus, Web of Science, PubMed and Embase a literature search was conducted to identify suitable original articles ( Table 1). MesH terms including “Radiomics”, “Machine learning”, “Meningioma”, “Outcome prediction”, “Recurrence”, “Overall survival analysis” along with Boolean operators like as “AND” “OR” (extended data). Only English language research with adult subjects having MRI examinations were included in the search criteria.

**Table 1.** Study retrieval method from database.

Database	Number of studies retrieved	Total
PubMed	53	157
Scopus	32	
Web of Science	32	
Embase	40	

### Selection criteria

**Inclusion criteria**

The articles which included the performance of ML models in the prediction of outcomes such as P/R and overall survival analysis for meningioma were included.

**Exclusion criteria**

Exclusion criteria include the ML based radiomics studies of other brain tumors, articles without radiomics or machine learning approaches. Articles included grading of meningioma, lacking outcome prediction were excluded.

### Data extraction

Two researchers independently reviewed each article, extracting details such as the authors name, publication year, sample size, tumour grades, classifiers used, and study outcomes. Any disagreements between the reviewers were settled by a third researcher.

### Quality assessment

The quality of the studies was assessed using Radiomics quality score (RQS) tool. ^20^ This tool assesses the ML based radiomic studies based on criteria with 16 items (underlying data) focusing on methodological rigor, reproducibility, and clinical relevance of radiomic studies.

## Results

**Figure 1.**
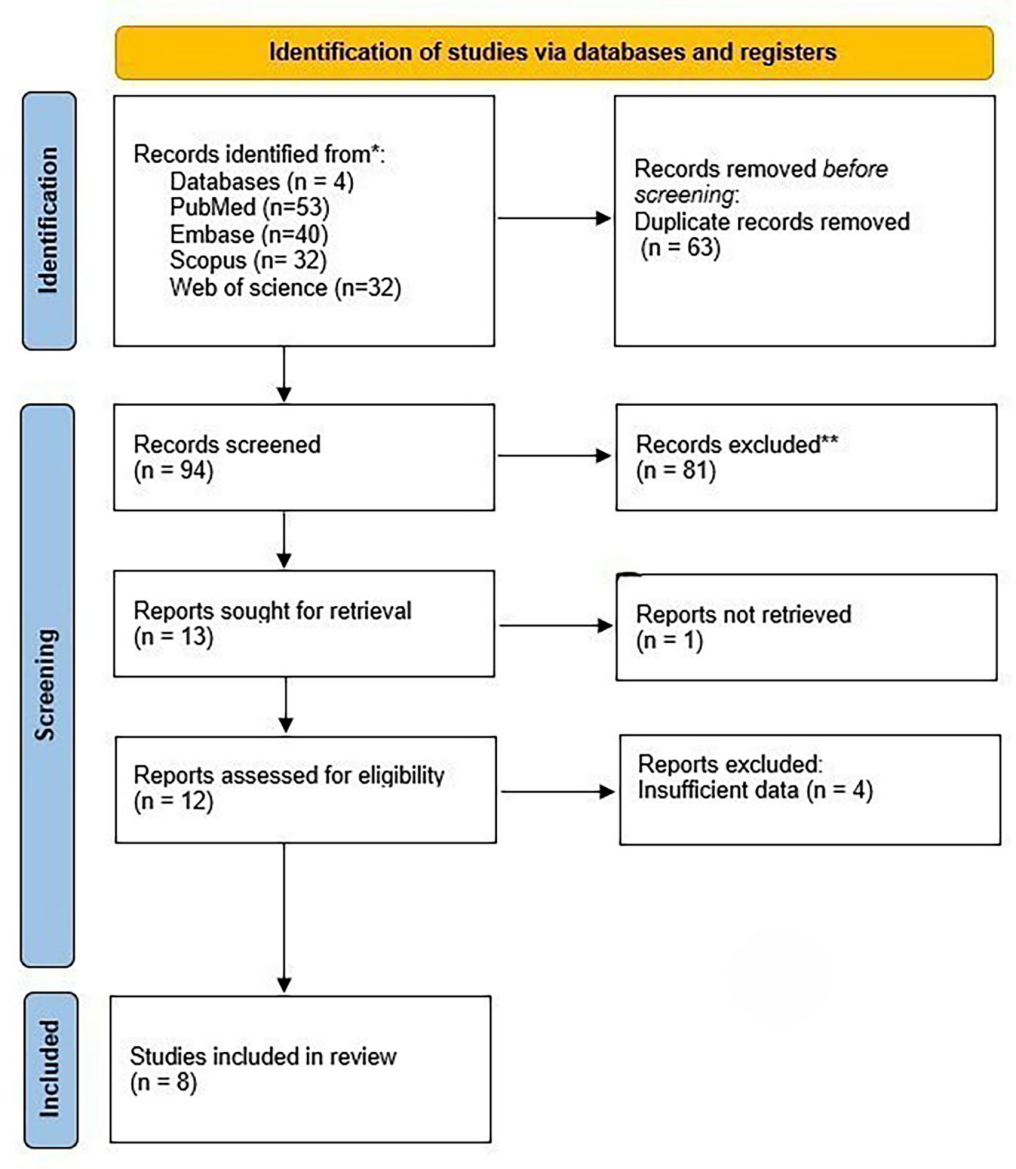
Flow chart for study selection.

A total of eight articles were included ( Figure 1).

### Study selection

157 studies were found in the early database search. The titles and abstracts of 94 articles were examined after 63 duplicates were removed, and 81 articles that did not fit the inclusion criteria were excluded. Twelve articles were left in the end. After evaluating the final text of these twelve articles for eligibility, four were disqualified for lacking adequate information such as comprehensive tables that measured the ML models performance. In the end, the final review contained eight articles.

### Characteristics of selected studies

The review encompasses research conducted in five different countries: Taiwan (n=3), China (n=2), South Korea (n=1), Germany (n=1), and the United States of America (n=1). A total of five studies utilized Siemens Healthcare MRI Scanners (Magnetom, Amira, Avanto, Aera, Sonata, and United Imaging 750) operating at field strengths of 1.5 T and 3 T, while three studies employed the GE Health Care MRI Scanner (Discovery MR 750) with a field strength of 3 T. The cumulative sample size across all studies amounted to 1237 participants. Eight articles included in the review followed retrospective analysis ( Table 2).

**Table 2.** showing the characteristics of selected studies.

S/No	Author & Year	Purpose	Sample size	Grade	Classifiers used	Outcome of the study
**Recurrence**						
1	Zhang et al. ^21^ 2019	Prognosis/recurrence	60 patients	I, II and III	Random forest algorithm	This study demonstrated that MR radiomic analysis significantly improves P/R prediction in SBM compared to traditional ADC-based methods. Preoperative radiomic assessments offer valuable insights for treatment planning, including tumor resection, adjuvant radiotherapy, and follow-up imaging schedules.
2	Ko et al., ^22^ 2021	Prognosis/recurrence	128 patients	I	SVM model	The SVM score from pre-operative MR radiomic features effectively predicted P/R in meningiomas. This approach offers crucial insights for treatment planning, including tumor resection, adjuvant radiotherapy, and follow-up imaging schedules.
3	Park et al., ^23^ 2022	Recurrence	155 patients	II	LASSO logistic classifier	Multiparametric MRI radiomics, combined with clinicopathological profiles, aids in predicting recurrence in atypical meningiomas. Radiomics identifies high-risk patients who may benefit from intensified treatment, making it a valuable tool for imaging grade 2 meningiomas.
4	Hsieh et al. ^24^ 2022	Prognosis/recurrence	57 patients	I	Light GBM, SVM, Random Forest classifier	ML-derived radiomic scores offer valuable insights to refine treatment strategies, including surgical resection, adjuvant radiotherapy, and MRI follow-up planning.
5	Ren et al. ^25^ 2024	Recurrence	224 patients	I, II and III	Cox Proportional Hazards Model	This study created a clinico-radiomics model (CRM) to predict recurrence in atypical meningiomas using clinical and MRI radiomics data. The CRM showed strong performance across validations and effectively identified high- and low-risk patients.
6	He et al. ^26^ 2024	Recurrence/prognosis	169 patients	I, II and III	LASSO and Cox Survival Analysis	The study developed an ML model combining clinicopathological data and MRI radiomics to predict P/R in meningioma patients, achieving an AUC of 0.88. The CPRM model aids personalized treatment by identifying high-risk patients, optimizing surgical and radiotherapy plans, and scheduling closer follow-ups.
7	Kalasauskas et al. ^27^ 2024	Recurrence	226 patients	I and II	Random Forest, SVM Neural Networks, Logistic Regression, AdaBoost Gradient Boosting, k-nearest-neighbours, Naïve Bayes	This study showed that radiomic analysis effectively assesses tumor aggressiveness and CNS WHO grade, outperforming evaluations by neuroradiologists and semantic feature scores.
**Overall survival**						
8	Morin et al. ^28^ 2019	Overall survival	218 patients	I, II and III	Random forest models	The study showed that combining clinical, radiologic, and radiomic features improved prediction accuracy for meningioma grade, local failure, and overall survival. Key imaging markers, such as ADC hypo-intensity and tumor sphericity, help identify high-risk, aggressive tumors.

SVM- Support Vector Machine, SBM- Skull base meningioma; GBM- Gradient Boost Machine, LASSO- Least Absolute Shrinkage and Selection Operator, P/R- Progression/recurrence, ML- Machine learning model, ADC- Apparent diffusion coefficient, CNS- Central nervous system, WHO- World health organization.

### Quality assessment

Due to the flaws in multiple segmentations, no Phantom studies, scanning at various times, cutoff analyses, calibration statistics, no prospective data collection, and cost effectiveness analysis, all of the studies received scores below 50% ( Table 3).

**Table 3.** Showing the radiomic quality scores of studies included.

Study & Year	Item 1	Item 2	Item 3	Item 4	Item 5	Item 6	Item 7	Item 8	Item 9	Item 10	Item 11	Item 12	Item 13	Item 14	Item 15	Item 16	RQS Total	RQS Percentage
Zhang et al. ^21^2019	+1	0	0	0	+3	0	0	+1	+1	0	0	+2	+2	+2	0	0	14	38.88%
Ko et al. ^22^ 2021	+1	0	0	0	+3	+1	+1	+1	+1	0	0	-5	+2	+2	0	0	7	19.44%
Park et al. ^23^ 2022	+1	0	0	0	+3	+1	+1	+1	+1	0	0	+2	+2	+2	0	0	14	38.88%
Hsieh et al. ^24^ 2022	+1	0	0	0	+3	+1	+1	+1	+1	0	0	+2	+2	+2	0	0	14	38.88%
Ren et al. ^25^ 2024	+1	0	0	0	+3	+1	+1	+1	+1	0	0	+2	+2	+2	0	0	14	38.88%
He et al. ^26^ 2024	+1	+1	0	0	+3	+1	+1	+1	+1	0	0	+2	+2	+2	0	0	15	41.67%
Kalasauskas et al. ^27^2024	+1	0	0	0	+3	+1	+1	+1	+1	0	0	+2	+2	+2	0	0	14	38.88%
Morin et al. ^28^ 2019	+1	0	0	0	+3	+1	+1	+1	+2	0	0	+3	+2	+2	0	0	16	44.44%

Summary of performance measures of various models of included studies were provided in ( Table 4).

**Table 4.** Showing the performance measures of various models across different studies.

S.No.	Author/Year	Model/ML with highest accuracy/validation dataset	AUC	Accuracy	F1 score	Sensitivity	Specificity	Recall	MCC
**Progression/Recurrence**									
1	Zang et al. ^21^ 2019	Radiomics + ADC correlation (Decision tree)	-	90	-	-	-	-	-
		ADC	0.88	83					
2	Ko et al. ^22^ 2021	Radiomics + ADC (SVM)	0.88	-	-	-	-	-	-
		SVM	0.80	-	-	-	-	-	-
		ADC	0.73	-	-	-	-	-	-
3	Park et al. ^23^ 2022	Clinicopathological	0.61	81.3	-	33.3	89.1	-	-
		Clinicopathological +radiomics	0.77	70.3	-	66.7	70.9	-	-
4	Hsieh et al. ^24^ 2022	Clinical Data	0.78	0.86	-	0.83	-	0.67	-
		Radiomic features	0.79	0.88	-	0.83	-	0.63	-
		Clinical+ Radiomics model (Random forest)	0.88	0.91	-	0.85	-	0.83	-
5	Ren et al. ^25^ 2024	Clinicopathological+ radiomics (External validation)	0.840	-		-	-	-	-
6	He et al. ^26^ 2024	Clinicopathological	0.817	0784		0.853	0.647	-	
		Combined radiomics	0.84	0.82		0.81	0.83	-	
		Clinicopathological radiomics	0.88	0.86		0.88	0.81	-	
7	Kalasauskas et al. ^27^ 2024	Radiomics (Random Forest)	0.892	88.2	0.750	-	-	-	0.717
		Semantic scores	0.65	-	-	-	-	-	-
		Radiological	0.54	-	-	-	-	-	-
**Overall survival analysis**									
8	Morin et al. ^28^ 2019	**Imaging models**							
		Radiologic	0.65	0.63	-	-	-	-	-
		Radiomics	0.75	0.67	-	-	-	-	-
		**Combined model**							-
		Radiological + radiomics	0.74	0.67	-	-	-	-	-
		Demographic + therapy + grade	0.71	0.64	-	-	-	-	-
		Demographic + radiologic +radiomic	0.77	0.7	-	-	-	-	-
		Demographic + radiologic +radiomic + therapy + grade	0.77	0.69	-	-	-	-	-

AUC- Area under curve; ADC: Apparent diffusion coefficient; MCC- Mathews correlation coefficient; SVM- Support vector machine.

### Performance measures of ML models for predicting meningioma recurrence and overall survival analysis

**For predicting meningioma P/R:**

A study by Zhang et al. ^21^ for predicting recurrence in skull base meningiomas (SBM), a radiomics-based decision tree model using preoperative MRI features demonstrated 90% accuracy, outperforming the apparent diffusion coefficient (ADC) model, which achieved accuracy of 83%. The most significant predictive features included T1 maximum probability, T1 cluster shade, and ADC correlation, selected via a random forest algorithm. The radiomics model identified 18 true positive, 36 true negative, 3 false positive, and 3 false negative cases, highlighting its strong predictive capability.

Ko et al. ^22^ for predicting progression/recurrence in meningiomas reported that a support vector machine (SVM) based radiomics model demonstrating strong predictive performance. The model, using preoperative MRI radiomic features, achieved an area under curve (AUC) of 0.80 with an optimal SVM score cutoff of 0.224. When combined with ADC, the predictive power improved to an AUC of 0.88. Multivariate analysis identified bone (Hazard ratio (HR):7.31, p<0.05), low ADC values (HR:4.67, p<0.05), and high SVM scores (HR:8.13,p<0.05) as significant risk factors for meningioma recurrence. Patients with higher SVM scores showed shorter progression-free survival (p=0.003), highlighting the potential of radiomics-based AI models for refining risk stratification and guiding treatment decisions.

Another study by park et al. ^23^ for predicting recurrence in WHO grade 2 meningiomas, the combined clinicopathological and radiomics model (CPRM) outperformed the clinicopathological model (CPM) alone. Compared to the CPM model (AUC:0.61, accuracy 81.3%, sensitivity: 33.3%), the combined clinic-pathological and radiomics model (CPRM) showed good performance with AUC:0.77, accuracy 70.03%, sensitivity 66.7% and specificity of 70.9%) in test data. This model also identified high-risk patients who significantly benefited from adjuvant radiotherapy (ART), with a 5-year progression free survival of 92.3% compared to 56.8% without ART.

A study by Hsieh et al. ^24^ for predicting recurrence in parasagittal and parafalcine meningiomas, the ML model combining clinical and MRI texture features showed the highest predictive performance. The study found that high radiomic scores (AUC:0.91), low ADC values (AUC:0.82), and large tumor diameter (AUC:0.69) were significant predictors of recurrence, and multivariate analysis showed that score cut off of 0.269 was a strong risk factor of early tumor recurrence with HR of 15.73 (p<0.05). This model outperformed models based on clinical data alone (AUC:0.78) and MRI texture features alone (AUC:0.79).

A study by Ren et al. ^25^ found that hybrid clinical-radiomics model that integrated radiomics features with clinicopathological data performed well in predicting recurrence in atypical meningiomas. In the training cohort, the model’s integrated AUC was 0.858 (0.802-0.915); in the internal validation cohort, AUC was 0.781; and in the external validation cohort it was 0.840. The ki-67 index, surgical history, radiomics signature, and degree of resection were all found to be independent predictors of atypical meningioma recurrence.

In a study by He et al. ^26^ the greatest predictive performance was shown by clinicopathological-radiomics model (CPRM) that combined clinicopathological characteristics with radiomic features from preoperative MRI to predict meningioma recurrence. With an AUC of 0.88 in the test set, the model outperformed the radiomics-only model (AUC 0.85) and the clinicopathological model (AUC:0.86). Key predictors included radiation history, Simpson grading, WHO grading, and ki-67 index.

Kalasauskas et al. ^27^ showed that for meningioma recurrence prediction, the best performing ML was random forest with information gain feature selection of 25 radiomic features. This model achieved an AUC of 0.892, accuracy of 88.2% (±10.84%), an F1 score of 0.750, and a Matthews correlation coefficient (MCC) of 0.717. While the support vector machine (SVM) model achieved a slightly higher AUC of 0.896, it had lower F1-score (0.182) and MCC (0.270), making random forest the preferred choice. Compared to conventional radiological assessment (AUC:0.53-0.54) and semantic feature scores (AUC:0.65), radiomics-based models demonstrated significantly superior predictive performance.

**For predicting overall survival analysis**

A study by Morin et al. ^28^ for predicting local failure and overall survival in meningiomas, an integrated prognostic model combining clinical, radiologic, and radiomic features demonstrated improved predictive performance. The model achieved an AUC of 0.78 for overall survival analysis (OS). Outperforming models based on clinical features alone. Key prognostic imaging-derived factors included ADC hypo intensity (HR:1.96, p=0.005), low sphericity (HR:2.0, p=0.02), multifocality (HR:2.13, p=0.003) and venous sinus involvement (HR:1.79, p=0.04).

## Discussion

Meningioma is the most prevalent brain tumor, primarily affecting adults, and it occurs with incidence of 7.86 per 100,000 people worldwide each year. ^1– 2^ MRI is the recommended imaging modality, and recurrence is associated with traditional imaging criteria such as tumor size and bone invasion. ^7– 9^ Radiomics, provides a non-invasive substitute for biopsies by evaluating tumor heterogeneity and identifying prognostic markers. Using machine learning to integrate radiomics with clinical and pathological data may improve the accuracy of diagnosis and prognosis of tumors. ^10– 12^ However there is lack of systematic review of ML-base radiomics models for meningioma survival and recurrence prediction.

Zhang et al. ^21^ and Kalasauskas et al. ^27^ reported the effectiveness of radiomics-based ML models in predicting tumor recurrence and aggressiveness in meningiomas. Using radiomic features from contrast enhanced T1Weighted image and ADC maps as primary predictors, Zhang et al. ^21^ created a random forest-based radiomics model that achieved 90% accuracy in predicting P/R in SBM. Their research showed that radiomics performed better than traditional MR imaging in objective and quantitative tumor categorization, offering useful prognostic information for decisions about surgery and treatment. Kalasauskas et al. ^27^ reported that radiomic analysis is more accurate than traditional neurological evaluation in predicting tumor recurrence and CNS WHO grade. The ML model performed well across heterogeneous MRI datasets, emphasizing the importance of the data preprocessing in lowering variability across varied MRI datasets. According to their study, preoperative evaluation based on radiomics may help with treatment stratification by directing regarding the extent of resection and follow-up imaging. Both the studies support the clinical promise of radiomics for better surgical planning, recurrence prediction, and tailored meningioma care.

The studies by Park et al., ^23^ Hsieh et al., ^24^ and Ko et al. ^22^ highlighted the role of radiomics-based ML models in predicting tumor recurrence in meningiomas and refining treatment strategies. Park et al. ^23^ demonstrated that integrating radiomic features with clinicopathologic data significantly improved recurrence prediction in grade 2 meningiomas. Their model identified high-risk patients who benefited from ART, supporting the use of radiomics for non-invasive risk stratification and personalized treatment decisions. Hsieh et al. ^24^ introduced a radiomic score-based approach, combining clinical and MRI texture features, achieving an AUC of 0.88 in predicting P/R in parasagittal and parafalcine meningiomas. Their study confirmed that integrating clinical and imaging data enhanced predictive accuracy compared to using either alone. Ko et al. ^22^ developed an SVM-based radiomics model, identifying adjacent bone invasion, low ADC values, and high SVM scores as key high-risk factors for recurrence. Their model outperformed traditional ADC-based assessments and emphasized radiomics’ potential in preoperative risk assessment. All three studies support radiomics as a valuable prognostic tool for identifying high-risk meningioma patients, optimizing surgical and ART decisions, and guiding personalized follow-up strategies.

The studies by Ren et al. ^25^ and He et al. ^26^ reinforce the predictive value of radiomics-based machine learning models in assessing tumor recurrence and progression-free survival (PFS) in meningiomas. Ren et al. ^25^ developed a clinicopathological-radiomics model (CPRM) incorporating radiomic features from T1Contrast and T2-FLAIR MRI alongside clinical factors, achieving an AUC of 0.858 for predicting recurrence in atypical meningiomas across multiple neurosurgical centers. Their model proved the utility of combining multimodal data for precision oncology, outperforming previous clinical and radiomics-only models.

He et al. ^26^ developed a complete clinicopathological-radiomics model (CPRM) that integrated three radiomics signatures (T1, eT1, T2), WHO grading, Simpson grading, Ki-67 index, and radiotherapy history, achieving AUCs of 0.93 for training dataset and 0.88 for testing dataset.

Their study produced a clinical normogram for personalized risk assessment and performed better than single-modality radiomics models (AUC:0.70-0.85). However, Simpson grading and Ki-67 remained the most significant predictors for progression free survival, suggesting that postoperative factors are crucial for long-term monitoring. Both studies highlighted the superior performance of integrated radiomics-clinicopathological models over traditional assessments, supporting their use in preoperative risk stratification and treatment planning for meningiomas.

The study by Morin et al. ^28^ highlighted the prognostic significance of radiologic and radiomic features in predicting meningioma grade, local failure (LF), and overall survival (OS). Their integrated model, combining preoperative MRI features with clinical variables, demonstrated significant predictive performance. ADC hypointensity and low tumor sphericity were identified as key radiomic predictors associated with high-grade meningiomas, poor local control, and reduced OS. Conventional radiological imaging features such as peritumoral edema, indistinct tumor margins, and absence of a CSF cleft sign, were associated with higher tumor grade and worse outcomes.

The review has few limitations. Firstly, meta-analysis was not conducted due to heterogeneity of data, including different MRI protocols and patient populations. Secondly, studies included had small sample sizes, limiting statistical power. Thirdly, most studies were retrospective, introducing potential biases.

## Conclusion

Our review suggests that radiomics greatly improved meningioma diagnostic and prognostic accuracy when combined with clinical and pathological data through machine learning. ML models based on radiomics performed better than traditional MRI in predicting tumor aggressiveness and recurrence, offering important information for surgical planning and individualized treatment. The combination of radiomics and clinicopathological data offers a non-invasive substitute for biopsies helping in risk stratification and guiding decisions on adjuvant treatment and follow-up plans. Overall, radiomics holds great potential for improving the management and outcomes of meningioma patients.

## Ethics and consent

Ethical approval and consent were not required

## Reporting guidelines

Fig share: https://doi.org/10.6084/m9.figshare.28557743.v1.

Data are available under the terms of the Creative Commons Attribution 4.0 International license (CC-BY 4.0).

## Data Availability

No data are associated with this article Figshare: Meningioma Recurrence. https://doi.org/10.6084/m9.figshare.28557743.v1. ^29^ This project contains the following extended data:
1. Supplementary File 1 (PRISMA_2020_check list) 2. Supplementary File 2 (MeSH terms used) 3. Supplementary File 3 (Radiomics quality score questions) 4. Supplementary File 4 (PRISMA Flow diagram)
