# Supplementary material for: Machine learning based radiomics approach for outcome prediction of meningioma – a systematic review
Source: F1000Res. 2025 Mar 25;14:330. [Version 1] doi: 10.12688/f1000research.162306.1 (PMC11979578; doi:10.12688/f1000research.162306.1)
Supplement: Peer review history

# Reviewer report: Reviewer response for version 1

**DOI:** 10.5256/f1000research.178486.r373394

**Published:** 2025-04-08

**Authors:** Mustapha Barde

**Affiliations:** 1 Bayero University, Kano, Kano, Nigeria

An impressive systematic review was conducted by the authors, to evaluate the performance of machine learning based radiomics approach in the prediction of meningiomas. It was reported that based on the studies been reviewed MRI based machine learning, based on decision tree models have more accuracy (90%) in the prediction of meningiomas reoccurrence. Surpassing apparent diffusion coefficient-based models (83%).  from MRI studies. It was reported that radiomics combined with clinicopathological data via Machine learning gives improved results in meningioma prediction compared to conventional MRI results. Also based on the review the meningioma survival analysis prediction, the radiomics and clinicopathological features attained an area under the curve of 0.78.

**General comment**. 

 The review is commendable. However, addressing the below minor corrections will enhance clarity and comprehension.

**Minor corrections** 

 The below points should be addressed please for clarity

 1. The total number of search articles from the databases should be stated in the abstract methodology, and the number excluded.

 2. In figure 1, an asterisk was used in records identified and records excluded. A key will guide in noting the rationale for the asterisk or ignoring the asterisk if not crucial.

 3. In the exclusion criteria, it was stated that articles that report grading were excluded. However, in Table 2. The grade column was included, with the relevant grading based on the studies reviewed. I suggest the exclusion criteria be restructured to align with the relevant search articles. It can be paraphrased as “also, articles lacking predictive outcome were excluded.”

 4. The study year in Table 3 should follow a uniform order (arrange in ascending order)

Are the rationale for, and objectives of, the Systematic Review clearly stated?

Yes

Is the statistical analysis and its interpretation appropriate?

Yes

If this is a Living Systematic Review, is the ‘living’ method appropriate and is the search schedule clearly defined and justified? (‘Living Systematic Review’ or a variation of this term should be included in the title.)

Not applicable

Are sufficient details of the methods and analysis provided to allow replication by others?

Yes

Are the conclusions drawn adequately supported by the results presented in the review?

Yes

Reviewer Expertise:

image analysis and processing

I confirm that I have read this submission and believe that I have an appropriate level of expertise to confirm that it is of an acceptable scientific standard.

# Reviewer report: Reviewer response for version 1

**DOI:** 10.5256/f1000research.178486.r373393

**Published:** 2025-04-07

**Authors:** Priyanka Chandrasekhar

**Affiliations:** 1 Department of Allied Health Sciences, The Apollo University, Chittoor, Andhra Pradesh, India

**Major comments:**

- The review article provides thorough information of machine learning (ML) based radiomic models for predicting meningioma outcomes. It effectively summarizes the performance measures of various models, highlighting their strengths and limitations.
- The results from eight studies were explained clearly. The comparison of different machine learning models was insightful.
- The quality assessment using the Radiomics quality score (RQS) is a strong point.
- The discussion highlighted the superior performance of ML models over traditional MRI predicting the tumor recurrence and aggressiveness, emphasizing their value in personalized treatment and planning.
- The conclusion effectively summarizes the findings and reaffirms the promise of integrating radiomics with clinical and pathological data in improving the management and outcomes of meningioma patients.

**Minor comments:**

- The article is well written, the sentence “Radiomics incorporates a range of advanced techniques used to conduct a comprehensive analysis of medical images” could be rephrased for clarity.

Are the rationale for, and objectives of, the Systematic Review clearly stated?

Yes

Is the statistical analysis and its interpretation appropriate?

Not applicable

If this is a Living Systematic Review, is the ‘living’ method appropriate and is the search schedule clearly defined and justified? (‘Living Systematic Review’ or a variation of this term should be included in the title.)

Not applicable

Are sufficient details of the methods and analysis provided to allow replication by others?

Yes

Are the conclusions drawn adequately supported by the results presented in the review?

Yes

Reviewer Expertise:

Advances in Magnetic Resonance Imaging , Artificial intelligence in Healthcare

I confirm that I have read this submission and believe that I have an appropriate level of expertise to confirm that it is of an acceptable scientific standard.
